# Benthic Diatom Based Indices for Water Quality Assessment in Two Subtropical Streams

**DOI:** 10.3389/fmicb.2017.00601

**Published:** 2017-04-07

**Authors:** Xiang Tan, Quanfa Zhang, Michele A. Burford, Fran Sheldon, Stuart E. Bunn

**Affiliations:** ^1^Key Laboratory of Aquatic Botany and Watershed Ecology, Wuhan Botanical Garden, the Chinese Academy of SciencesWuhan, China; ^2^Australian Rivers Institute, Griffith UniversityNathan, QLD, Australia

**Keywords:** benthic algae, biological monitoring, community, diatom index, microbial, periphyton

## Abstract

Benthic diatoms have been universally used as indicators to assess water quality in lotic ecosystems. However, most diatom-based indices developed in Europe have not been widely used or tested in other continents such as Asia or Oceania. This study compared the performance of 14 widely-applied diatom indices in assessing ecological conditions in subtropical streams in South East Queensland (SEQ) in Australia and in the upper Han River in China. Most water quality variables in the upper Han River including dissolved organic carbon (DOC), total nitrogen (TN), and soluble reactive phosphorus (SRP) had strong relationships with at least one diatom index, with the exception of IDAP (Index Diatom Artois-Picardie), and TDI (Trophic Diatom Index). However, in SEQ, most of the environmental variables including DOC, ammonia nitrogen (NH_4_-N), TN, SRP, and electrical conductivity (EC) showed no significant relationships with diatom indices, and the DI-CH (Swiss Diatom Index) and WAT (Watanabe's Index) were unrelated to any of the variables examined. Only pH and nitrite or nitrate nitrogen (NO_X_-N) were significant predictors of several diatom indices in SEQ, especially TID (Rott trophic index). In the upper Han River, much of the spatial variation in most diatom indices was explained by proximate determinants alone, including EC, DOC, dissolved oxygen (DO) or SRP, or a combination of ultimate (canopy, forest) and proximate factors (*R*^2^ in most models> 0.75). Most diatom indices performed as predicted in the upper Han River where nutrient and organic matter pollution was relatively high, and variation in pH low. However, the indices performed poorly in SEQ where the water quality gradient was low and instead most responded to spatial variation in pH. This finding serves as a caution to the application of diatom indices in river basins that fall outside of the range of water quality values of the systems in which they originally developed.

## Introduction

River ecosystems are under threat from various human activities across the globe leading to considerable changes in sediment delivery and flow patterns, declining water quality and loss of biodiversity (Dudgeon et al., 2006; Vörösmarty et al., 2010). Many streams are heavily impacted by land use change for agriculture and urban development (Allan, 2004) and some regions are under increasing pressure from rapid development (e.g., Weihoefer and Pan, 2006; Bunn et al., 2007). For example, human activities including agriculture and urban areas increasement resulted in alterations in watershed hydrology and sediment delivery, water quality deterioration in South East Queensland (Bunn et al., 2007). The major anthropogenic disturbances in Oregon Coast Range which is under increasing pressure associated with forest management practices and probably lead to declining status of stream biota such as salmonid fish (Weihoefer and Pan, 2006). With increasing degradation of freshwater ecosystems worldwide, there is a growing demand for effective approaches to ecosystem condition monitoring and evaluation, and bioassessments have been implemented in many countries.

Diatoms have been recognized as good indicators of land use change and water quality (Chessman et al., 2007; Chessman and Townsend, 2010; Lavoie et al., 2014; Stevenson, 2014). They represent an important component of freshwater ecosystems and respond quickly to environmental change. Several diatom indices have been implemented around the world. Examples include IPS (CEMAGREF, 1982), Trophic Diatom Index (TDI, Kelly and Whitton, 1995) and the Diatom Biological Index (IBD, Coste et al., 2009), which are based on a weighting average equation. There are also multimetric indices including the Biotic Integrity Index, which uses diatom community structural metrics based on relative abundance (Wang et al., 2005). Diatom indices developed in Europe have been confirmed for successful application in other temperate regions, however, there is little information regarding their suitability for assessing water quality in subtropical or tropical zones (Taylor et al., 2007).

There have been few studies on developing specific diatom indices for ecological health assessment in aquatic ecosystems in Australia and China (Chessman et al., 2007; Tan et al., 2013, 2014a, 2015). Our aim was to compare the effectiveness of diatom based indices used worldwide in subtropical streams in these two contrasting regions. Specifically, our aims were to: (1) determine the response of benthic diatom-based indices to proximate (e.g., flow velocity and nutrients) and ultimate environmental factors (e.g., land use and canopy cover in riparian zone); and (2) compare the robustness of the diatom indices in subtropical rivers in detecting environmental gradients between these two different geographic regions, i.e., whether they respond to stressors in the same ways.

## Methods

### Study area

**Figure 1:**
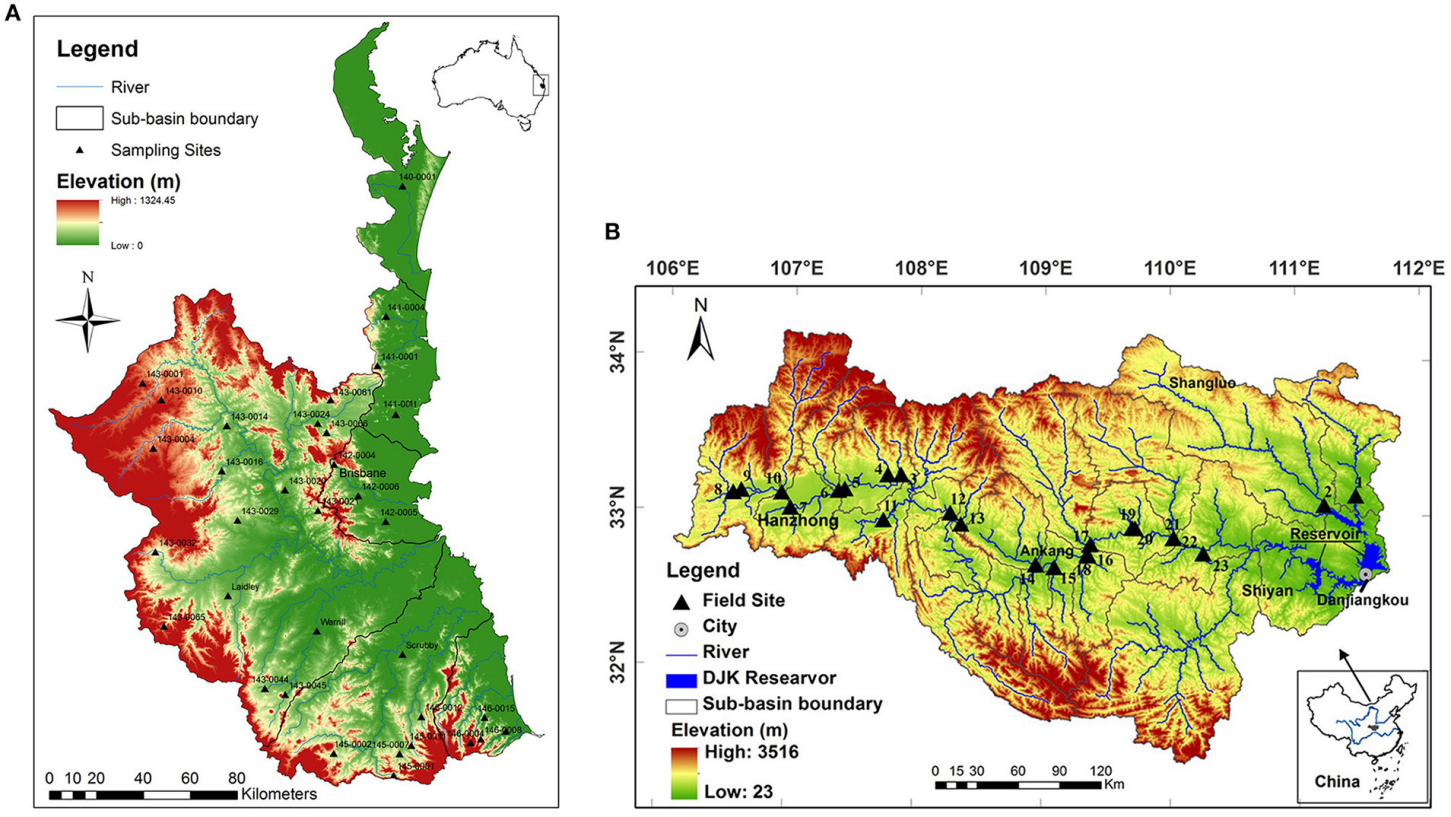
**The location of sampling sites in streams in South East Queensland (A)** and in the upper Han River basin **(B)**.

The South East Queensland (SEQ) region with an area of 22,672 km^2^ is located in the subtropical climate zone and is the fastest developing area in Australia (Figure 1A; Abal et al., 2005). The region covers 15 major catchments including the upper Brisbane River, Lockyer Creek, and the Bremer, Logan, and Albert Rivers, which drain into Moreton Bay and the Pacific Ocean. The most dominant land use in the upper catchments is cattle grazing (Kerr et al., 2011). The other area of study, the Han River, is one of the first order tributaries of the Yangtze River (Changjiang) with a length of 1,577 km. The upper Han River basin is defined as a watershed upstream of the Danjiangkou Reservoir (Figure 1B). The drainage area is 95,200 km^2^ with a length of 925 km for the mainstream of the upper Han River. Agricultural land accounts for about 15% of total land area. Further information on the studied watersheds can be found in Tan et al. (2014a).

In SEQ subtropical streams the average TN concentration was about 1.0 mg L^−1^ (Haase and Nolte, 2008), which is considerably lower than that reported in some subtropical rivers in China (Li et al., 2009; Tan et al., 2014a). In the upper Han River, nitrate nitrogen (NO_3_-N) varied from 0.2 to 15.3 mg L^−1^ with an average of 1.6 mg L^−1^; NH_4_-N varied from 0.04 to 6.99 mg L^−1^ with an average 0.3 mg L^−1^ (Li et al., 2009). In SEQ, about half of the 48,000 km of streams have degraded riparian zones (Bunn et al., 2010), though the percentage of dense riparian forest cover upstream of our sites ranged from 0 to 51% with an average of 12% (Tan, 2015). In contrast, the average of the remaining riparian zone vegetation cover was only 2% in the upper Han River except for that along some headwater creeks (Tan et al., 2014b).

### Physical and chemical parameters

Water samples were taken from the 34 sites in SEQ in October 2011 and 23 sites in the upper Han River in April 2010 (both in spring) (Figure 1). Temperature (*t*), EC, DO, pH, and turbidity were measured *in situ* using a Hydrolab Quanta Multiparameter meter. The velocity of flowing waters was detected using a current velocity meter (Model 2100, Swoffer Instruments, INC). Triplicate samples of the surface water at each site were collected and filtered using cellulose nitrate membrane filters (Whatman, 0.45 μm pore size) for the analysis of total dissolved nutrient such as dissolved nitrogen (TDN), SRP, NH_4_-N, nitrite or nitrate nitrogen (NO_X_-N), and DOC by standard methods. Triplicate water samples were also collected for TN and total phosphorus (TP) analysis. Samples for analysis of dissolved nutrients were stored on ice in a cooler, and then frozen before analysis. Samples for SRP, NH_4_-N, and NO_X_-N concentrations were analyzed with colorimetric assays using a Discrete Chemistry Analyser (SmartChem200, Westco Scientific Instruments Inc., Brookfield). The samples for total nutrients were digested in the laboratory using a simultaneous persulfate digestion method (Hosomi and Sudo, 1986) and were then analyzed colorimetrically using a flow injection analyser (LACHAT 8000QC). DOC and TDN were measured using a TOC/TN analyser, which was equipped with different modules for measuring TOC and TN (Shimadzu Corporation).

### Epilithic diatom sampling and identification

Epilithic diatoms were sampled by randomly selecting 5 cobbles within riffles of the sampling reach. An area with a diameter of 40 mm from each rock was scraped using a toothbrush and combined into 1 composite sample of 100 ml per site. Two composite samples for algae identification were conducted at each site. Samples for diatom identification and enumeration were preserved with formaldehyde with a final concentration of formaldehyde of 4% in the samples.

For diatom slide preparation, samples were rinsed with deionized water to remove the formaldehyde and then digested with sulfuric acid (H_2_SO_4_) followed by nitric acid (HNO_3_). Samples were rinsed repeatedly with deionized water until the pH was approximately neutral and then mounted with Naphrax (Weihoefer and Pan, 2006). Benthic diatoms were mounted with Naphrax™ and were identified at 1,000× magnification (Weihoefer and Pan, 2006; Tan et al., 2013). A minimum of 400 valves was counted per slide at 1,000× magnification. The methods for determinations of the algal taxonomy have been described by Tan et al. (2013).

### Land use and canopy cover percentage analysis

To estimate land use and canopy cover in SEQ, Landsat TM imagery was obtained from the work undertaken by Peterson et al. (2011). The Statewide Landcover And Trees Study (SLATS) Foliage Projective Cover data was used for the classification of forest (Armston et al., 2009). The urban data are from the National Environmental Stream Attributes (https://www.ga.gov.au/products/servlet/controller?event=GEOCAT_DETAILS&catno=73045). Land cover types such as urban, mid-dense forest and dense forest were quantified using ArcGIS 10.1 Desktop GIS software.

Land cover classes in the upper Han River were categorized into five major classes, i.e., vegetation (forest, shrub), agriculture, urban, water surface and bare lands. The sampling site coordinates were used as the outlet point for each watershed and each land cover class was displayed as a percentage in its respective total subcatchment (Weihoefer and Pan, 2006). Land use percentage in the upper Han River was quantified using Landsat-7 ETM^+^ (2012) with supervised classification algorithms with ArcGIS 10.0 and ERDAS IMAGING 9.2 software.

In both regions, images of the riparian canopy were taken at 50–100 cm above the water surface at the spot where the samples were collected using a Nikon digital camera and fish-eye lens. Digital images were then analyzed for canopy cover percentage using Gap Light Analyser (GLA), Version 2.0.

### Data analysis

Fourteen diatom based indices (Table 1, Tan et al., 2013), which have been widely applied for assessment of ecological conditions, were calculated using OMNIDIA 7 software V 4.2 (Lecointe et al., 2003). Initially, the relationships between the diatom based indices and measured environmental factors (ultimate and proximate) in each study area were explored using Pearson's correlations. As there were multiple comparisons, the significance was Bonferroni-corrected and only assumed where *p* < 0.01. For each diatom index, stepwise multiple regression was then used to explore which combination of ultimate and proximate variables best explained the observed variation in the diatom index (Tan et al., 2013). All statistical analyses were performed using IBM SPSS statistics for windows (IBM Corp, Version 21.0. Armonk, NY: IBM Corp.).

**Table 1:** **Diatom based indices and the acronyms used in this study (from Tan et al., 2013, all the references in the Table can be found in Tan et al., 2013)**.

**Abbreviation**	**Diatom indices**
CEE	Commission for Economical Community metric (Descy and Coste, 1991)
DESCY(or ID)	Descy's pollution metric (Descy, 1979)
DI-CH	Swiss Diatom index (Lecointe et al., 2003)
EPID	Diatom-based eutrophication/pollution index (Dell'Uomo, 1996)
IBD	Biological Diatom index (Prygiel and Coste, 2000)
IDAP	Index *Diatom* artois-picardie (Prygiel et al., 1996; Lecointe et al., 2003)
IDP	Pampean diatom index (Gómez and Licursi, 2001)
IPS	Specific pollution sensitivity Index (CEMAGREF, 1982)
SHE	Schiefele and Schreiner's Index (Schiefele and Schreiner, 1991)
SID	ROTT saprobic index (Rott et al., 1997)
SLAD	Sládeček's index (Sládeček, 1986)
TDI	Trophic Diatom index (Kelly et al., 1995)
TID	ROTT trophic index (Rott, 1999)
WAT	Watanabe's Index (Watanabe et al., 1986; Lecointe et al., 2003)

## Results

### The epilithic algae assemblage composition

In SEQ, the Bacillariophyta (diatoms) comprised 86.4% of the total taxa. There were a total of 198 Bacillariophyta species from 48 genera (Appendix 1). The genera *Nitzschia, Navicula*, and *Gomphonema* accounted for the most species with 38, 30, and 15 species, respectively.

In the upper Han River, the Bacillariophyta comprised about 90% of the total taxa. The genera *Achnanthidium, Fragilaria*, and *Cymbella* were the most common genera with an average abundance of 35, 24, and 19% in epilithic communities, respectively. The number of taxa found (132 taxa from 33 genera of Bacillariophyta) (Appendix 2) was smaller than the 198 taxa from SEQ streams. Of the taxa in both catchments, *Gomphonema minutum* Agardh was the most common species, with a relative abundance of 12.5% in SEQ and 5.9% in the upper Han River (Table 2). For further details about the epilithic diatom community composition, see Tan (2015).

**Table 2:** **The dominant species and their relative abundance (RA) in South East Queensland (SEQ) and the upper Han River (uHR) (relative abundance refers only to the Bacillariophyta; site number is 34 in ***SEQ*** and 23 in ***uHR***)**.

***SEQ***		***uHR***	
**Dominant species**	**RA (%)**	**Dominant species**	**RA (%)**
*Rhoicosphenia abbreviata* (Agardh) Lange-Bertalot	15.60	*Achnanthidium pyrenaicum* (Hustedt) Kobayasi	17.10
*Gomphonema minutum* (Agardh) Agardh	12.50	*Achnanthidium subatomus* (Hustedt) Lange-Bertalot	12.50
*Cocconeis placentula* var.*euglypta* (Ehrenberg) Grunow	9.60	*Achnanthidium saprophila* (Kobayasi & Mayama) Round & Bukhtiyarova	6.40
*Achnanthidium minutissimum* (Kützing) Czarnecki	7.00	*Gomphonema minutum* (Agardh) Agardh	5.90
*Gomphonema* sp.	6.20	*Melosira varians* Agardh	5.80

### The diatom indices

In SEQ, five diatom indices (CEE, DESCY, SID, SLAD, and TID) were significantly correlated with one or more environmental variables in the streams after Bonferroni correction (Table 3). Only canopy cover in the riparian zone and pH were significantly correlated with these indices. Among the 14 diatom index regression models (stepwise regression models), 70% of the observed variation in TID was explained by pH together with NO_X_-N (Table 4). Some indices appeared to be responsive to ultimate factors such as the percentage of canopy vegetation cover in the riparian zone or upstream urban areas in the catchment. Variation in DESCY could be explained by the percentage of canopy cover in riparian zones; IBD was predicted by the percentage of urban area upstream in the catchment and IDAP by the percentage of forest cover.

**Table 3:** **Pearson correlation coefficients between measured environmental variables and diatom indices in South East Queensland (***SEQ***)(a) and the upper Han River (***uHR***)(b)**.

	**Urban**	**Forest**	**Canopy**	**Velocity**	**DOC**	**NH_4_-N**	**NO_X_-N^a^/ NO_3_-N^b^**	**TN**	**TP**	**SRP**	***t***	**EC**	**pH**	**Turbidity**
***SEQ***														
CEE							0.368		−0.377				−0.524^**^	
DESCY			0.462^**^										−0.379	
DI-CH														
EPI-D													−0.409	0.403
IBD							0.352						−0.371	0.362
IDAP														0.434
IDP											−0.433			
IPS													−0.420	0.387
SHE														0.363
SID			0.410										−0.533^**^	
SLAD													−0.525^**^	
TDI													0.669^**^	
TID				0.366			0.355						−0.587^**^	0.439
WAT														
***uHR***														
CEE			0.576^**^		−0.449	−0.566^**^		−0.532		−0.637^**^			0.483	
DESCY				0.446				−0.470		−0.541				
DI-CH	−0.680^**^		0.538	0.538	−0.638^**^			−0.524		−0.552		−0.552		
EPID			0.686^**^	0.455	−0.524	−0.492		−0.473		−0.579^**^		−0.446	0.575^**^	
IBD			0.565^**^	0.605^**^	−0.447	−0.475		−0.500				−0.507	0.622^**^	
IDAP					−0.461									
IDP	−0.474									−0.456				
IPS		0.469	0.534	0.604^**^	−0.585^**^	−0.582^**^		−0.599^**^		−0.468		−0.572^**^	0.562^**^	
SHE	−0.717^**^		0.537	0.556	−0.645^**^			−0.510		−0.540				
SID	−0.701^**^		0.582^**^		−0.625^**^					−0.512	−0.512			
SLAD			0.638^**^							−0.551			0.675^**^	
TDI			−0.522											
TID		0.582	0.679^**^	0.470	−0.547	−0.445				−0.465		−0.444	0.605^**^	
WAT	−0.829^**^		0.460	0.506	−0.551			−0.510						

** *p < 0.01; significance at the probability level marked in italics and assumed significant after Bonferroni correction (Blank means no significant correlation)*.

**Table 4:** **Predictive models with diatom indices as dependent variables using stepwise multiple regression**.

**Dependent variable**	**Predictors**	**Formula**	***R* square**	***F***	**Significance**				
					**Overall**	**Constant**	**Var. 1**	**Var. 2**	**Var. 3**
***SEQ***									
CEE	pH, NO_X_-N	26.5-2.2 pH+11.0 NO_X_-N	0.45	12.19	0.000	0.000	0.000	0.004	
DESCY	Canopy, NH_4_-N, pH	18.1+0.03 Canopy+16.9 NH_4_-N-0.9 pH	0.45	7.84	0.004	0.000	0.005	0.004	0.038
DI-CH	−	−	−	−	−	−	−	−	−
EPI-D	pH, Turbidity	20.8-1.4 pH +0.1 Turbidity^−^	0.29	6.16	0.006	0.000	0.026	0.030	
IBD	pH, NO_X_-N,Urban	22.2-1.9 pH+16.9 NO_X_-N+0.2 Urban	0.37	5.70	0.003	0.000	0.042	0.009	0.043
IDAP	Turbidity, Forest	7.7+0.2 Turbidity+0.05 Forest	0.39	9.63	0.001	0.000	0.001	0.003	
IDP	*t*	15.7-0.2*t*	0.17	6.17	0.019	0.000	0.019		
IPS	pH, NO_X_-N	25.0-2.1 pH+12.3 NO_X_-N	0.29	6.23	0.005	0.000	0.009	0.030	
SHE	Turbidity	11.5+0.06 Turbidity	0.13	4.77	0.037	0.000	0.037		
SID	pH, NO_X_-N	20.9-1.1 pH+4.6 NO_X_-N	0.37	8.68	0.001	0.000	0.001	0.047	
SLAD	pH	45.0-1.5 pH	0.29	12.35	0.001	0.000	0.001		
TDI	pH	−16.4+4.1 pH	0.45	25.69	0.000	0.008	0.000		
TID	pH, NO_X_-N	22.9-2.2 pH+10.2 NO_X_-N	0.70	15.47	0.000	0.022	0.000	0.005	
WAT	−	−			−	−	−	−	−
***uHR***									
CEE	SRP, TN, Forest	13.74-110.04 SRP-0.29 TN+ 0.06 Forest	0.77	23.2	0.000	0.000	0.001	0.005	0.038
DESCY	DOC	16.60-8.63 DOC	0.46	8.81	0.024	0.000	0.004		
DI-CH	DOC, Canopy	12.52-14.56 DOC+0.35 Canopy	0.83	25.90	0.002	0.000	0.000	0.005	
EPI-D	Canopy, DOC	11.39+0.55 Canopy-8.40 DOC	0.67	15.89	0.001	0.000	0.003	0.025	
IBD	Canopy, Velocity	13.3+0.4 Canopy+ 3.9 Velocity	0.62	10.90	0.004	0.000	0.012	0.026	
IDAP	−	−	−		−	−	−	−	−
IDP	NO_3_-N	12.9-0.2 NO_3_-N	0.27	5.80	0.005	0.000	0.038		
IPS	EC, SRP, Urban	22.8-0.02 EC-85.6SRP-0.8 Urban	0.82	20.83	0.000	0.000	0.000	0.001	0.005
SHE	Urban, SRP,	15.2-1.4 Urban-77.5 SRP	0.75	22.45	0.000	0.000	0.000	0.001	
SID	DOC, Forest	9.9-11.1 DOC+0.1 Forest	0.80	13.21	0.000	0.000	0.000	0.003	
SLAD	pH, SRP	−28.1+5.3 pH-61.4 SRP	0.80	13.13	0.000	0.004	0.000	0.003	
TDI	Canopy	12.0-0.6 Canopy	0.26	5.70	0.030	0.000	0.004	0.039	
TID	Canopy, NH_4_-N	7.8+0.4 Canopy-1.0 NH_4_-N	0.68	16.12	0.000	0.000	0.000	0.004	
WAT	Urban, SRP	17.0-2.2 Urban-67.5 SRP	0.79	27.52	0.000	0.000	0.000	0.030	

In uHR, most diatom indices had a strong correlation with one or more environmental variables, once Bonferroni corrected (Table 3). Except for temperature, NO_3_-N and turbidity, most water quality variables had strong relationships with at least one diatom index. A larger proportion of the observed variation in diatom indices was explained by environmental variables in the uHR compared with SEQ (Table 4). A significant portion of the observed variation in DESCY, DI-CH, EPI-D, IPS, and SID was explained by DOC, while variation in SHE and WAT were primarily explained by nitrogen concentration (NO_3_-N) (Table 4). Much of the observed variation in CEE, DI-CH, IPS, SHE, SID, SLAD, and WAT was explained by proximate determinants such as EC, DOC, DO, and SRP or by the combination of ultimate factors (i.e., canopy % in riparian zone, forest % in the upstream) and proximate factors (*R*^2^ > 0.75). Land use upstream (forest or urban land cover) explained a significant proportion of the variation in three indices, CEE, SHE, and SID.

## Discussion

These results indicate that the diatom indices can be used as bioindicators of anthropogenic activities such as land use change, riparian zone degradation, and nutrient loading, however, their performance varied markedly between the two regions. There is general agreement that a good indicator will consistently detect change across space and time, but will be sufficiently sensitive to respond to changes in environmental factors (Cottingham and Carpenter, 1998; Nelson et al., 2013). The sensitivity of diatoms has been confirmed by the significant relationships between measured proximate environmental variables and diatom indices in this study (Table 3). Furthermore, the influence of ultimate factors such as land use and land cover on diatom indices was also evident. Other studies have shown that not only the spatial pattern of epilithic diatom assemblages but also the diatom indices respond to ultimate factors such as land use change (Leland and Porter, 2000; Pan et al., 2004). The reason may be that the effects of human activities on landscape, such as conversion of forest to agricultural area, or degradation of riparian zone canopy have directly or indirectly influenced water quality parameters in waterways (Leland and Porter, 2000; Leland et al., 2001; Snyder et al., 2002; Potapova and Charles, 2003; Carr et al., 2005).

Diatom indices have been developed because they have been found to respond to water quality gradients including nutrients (Kelly et al., 1995), ion concentrations (Prygiel et al., 2002), and organic loading (e.g., WAT, Watanabe et al., 1986). However, this study has showed that few indices respond in the way as they were predicted and indeed some responded to other environmental factors.

Why do the diatom indices respond to disturbance differently in two basins? There are several possible explanations. First, the prediction of diatom indices may depend heavily on the similarity of species composition in the area of interest and the taxa used for developing each index. For example, IDP (Pampean Diatom Index) was not correlated with any environmental parameters in SEQ probably because only 33 taxa (16% of the total 203 identified species) were included in the taxa list (210 species) in the IDP calculation (Gómez and Licursi, 2001). In SEQ, WAT did not perform well because only a small proportion of taxa were included in the taxa list (548 diatom taxa) used in the WAT calculation (Watanabe et al., 1986). Also, IDAP cannot be explained by any environmental variables in the uHR, which is consistent with the findings in the same region in November 2007 (Tan et al., 2013). In SEQ, 70% of variation in TID was explained by pH and to a lesser degree NO_X_-N. The TID (Rott trophic index) is predicted to respond to environmental variables which relate to trophic state including nitrogen levels (Rovira et al., 2012). The majority (>70%) of species which were included in the development of the TID index were also found in our studies.

The IPS (Specific Pollution Sensitivity Index) has been regarded as one of the most precise indices to calibrate other indices because it incorporates approximately 2000 species, the largest among all diatom indices (Descy and Coste, 1991). More than 70% of the common species in the uHR could be found in the IPS list. Most of the variation (82%) in the IPS index was explained by parameters such as EC, SRP concentration and urban area% in the uHR, which is consistent with the finding that IPS responds to water quality parameters related to conductivity and eutrophication (Descy and Coste, 1991). These examples support the view that the performance of diatom-based indices in part depends on degree of overlap between the taxa list in the index development and those that occur in the sampled streams.

Another issue likely to affect the performance of diatom indices in different geographic regions is the range in water quality in the region where the diatom index was originally developed and the range in the regions where it is used. Among diatom communities, the relative importance of an environmental variable in accounting for variation depends on its range of variation in the data set (Potapova and Charles, 2002). The range of ${\text{PO}}_{4}^{3-}$ in the original region where the IDP developed was from 0.46 to 6.92 mg L^−1^ (Gómez and Licursi, 2001). However, the range of SRP in SEQ varied from only 0.00 to 0.16 mg L^−1^ with an average of 0.03 mg L^−1^, while the concentration of TP varied from 0.01 to 0.18 mg L^−1^ with an average of 0.05 mg L^−1^. Similarly, in the uHR the range of SRP varied from 0.007 to 0.06 mg L^−1^ and the concentration of TP varied from 0.02 to 0.4 mg L^−1^ (Tan et al., 2013, 2014a,b). The average of TN concentration was 2.5 mg L^−1^ in rivers in Poland where IBD and IPS were tested (Szulc and Szulc, 2013). In comparison, the average of TN concentration in SEQ rivers was only 0.06 mg L^−1^, which was approximately one fortieth that of the rivers in Poland. Similarly, the average of SRP was 0.2 mg L^−1^ in Polish rivers (Szulc and Szulc, 2013) but only 0.03 mg L^−1^ in SEQ. Obviously, the range of nutrients in SEQ is different from the range in North-Central European freshwater systems where most indices have been developed.

It is not surprising that environmental parameters that can explain most of the variation of diatom indices such as IPS in SEQ are different from the parameters in the uHR. For example, IPS was correlated with parameters related to organic pollution, ionic strength, and eutrophication of water quality (Descy and Coste, 1991). It was found that IPS significantly correlated with nitrate in the Artois-Picardie water Basin in France (Prygiel and Coste, 1993). The environmental variables associated with the diatom indices probably differ across geographic regions because the limiting factors for reproduction and growth are different.

Finally, it is very interesting that, in contrast to many other streams/rivers in the world (Gomà et al., 2005; Kalyoncu et al., 2009; Tan et al., 2013), diatom indices in SEQ responded more strongly to pH than to nutrients. Although diatom indices are normally used to assess the trophic state of waters, diatoms are sensitive to pH and liable to respond if there is a large gradient in pH across a broad scale (Potapova and Charles, 2002). For example, CEE, DESCY IPS, and SLAD have been found to correlate with pH (Prygiel and Coste, 1993; Prygiel et al., 2002). The pH gradient in streams in SEQ was large, from 5.36 to 8.12, but the nutrient gradient was small (Table 5). Compared with previous studies (Gomà et al., 2005; Kalyoncu et al., 2009), water conditions in SEQ are quite different from other regions in the world where these indices have been developed and tested. According to the literature, diatom indices have been explored in few rivers with the same combination of large pH gradient and low nutrients as in streams in SEQ. The streams in Argentina (Gómez and Licursi, 2001) where IDP index was developed had a similar pH gradient to that in SEQ but also had a high nutrient gradient.

**Table 5:** **Summary of the nutrient and pH range where the diatom indices perform well in the world**.

**Diatom index**	**pH range-small (range ≤ 2)**	**pH range-medium (<2 range <3)**	**pH range-large (range ≥3)**
Nutrient range-small (TN: range ≤ 0.1 mg L^−1^; TP or SRP: range ≤ 0.02 mg L^−1^)	IBD in the River Loup in Alpes Maritimes, France (Prygiel et al., 2002).		14 diatom indices in SEQ (this study).
Nutrient range-medium (TN:0.1 mg L^−1^ < range <5 mg L^−1^; TP or SRP: 0.02 mg L^−1^ < range <0.2 mg L^−1^)	EPI-D, IDAP, IPS in coastal streams in the Gulf of Gdansk Region (Zgrundo and Bogaczewicz-Adamczak, 2004); CEE, IBD and IPS in the upper Segre basin (La Cerdanya, Oriental Pyrenees) (Gomà et al., 2005).	EPI-D, TDI and IPS in waters in wetlands of central Italy (Bella et al., 2007).	
Nutrient range-large (TN: range ≥5 mg L^−1^; TP or SRP: range > 0.2 mg L^−1^)	IPS and TDI in England and Scotland (Kelly et al., 1995); DI-CH in South Turkey (Kalyoncu et al., 2009); EPI-D, IBD and IPS in the upper Segre basin (La Cerdanya, Oriental Pyrenees) (Gomà et al., 2005); EPI-D, IBD and WAT in the uHR, China (Tan et al., 2013).	EPI-D, TDI and IPS in waters in wetlands of central Italy (Bella et al., 2007).	IDP inrivers and streams in Argentina (Gómez and Licursi, 2001).

## Conclusions

This study has shown that diatom indices respond to ultimate environmental factors such as land use change and proximate factors including water quality across two subtropical basins. One diatom index (TID) in SEQ and 10 diatom indices in the upper Han River performed well (above 50% of the observed variation in the diatom indices was explained by environmental variables). More diatom indices performed well in the China streams than in the Australian streams because there was more overlap in the range of water quality variables between China and the regions where the indices were developed. There was also less of an overlap in diatom species between the original regions of the indices (Europe) and Australia compared with China. In the absence of a strong pollution gradient, diatom indices in SEQ responded strongly to natural variations in pH.

## Author contributions

XT, QZ, FS, and SB conceived and designed this study. XT performed the field trip, sample analysis and data analysis. XT drafted the original manuscript. MB provided comments. SB provided the very constructive suggestion and revision.

### Conflict of interest statement

The authors declare that the research was conducted in the absence of any commercial or financial relationships that could be construed as a potential conflict of interest.
