# Supplementary material for: Benthic Diatom Based Indices for Water Quality Assessment in Two Subtropical Streams
Source: Front Microbiol. 2017 Apr 7;8:601. doi: 10.3389/fmicb.2017.00601 (PMC5383657; doi:10.3389/fmicb.2017.00601)
Supplement: Supplementary file 1 [file DataSheet1.DOCX]

Appendix 1 List of diatom species in South East Queensland

(SEQ), Australia in October 2011.

| *Achnanthes brevipes* Agardh |
| --- |
| *Achnanthes clevei* Grunow |
| *Achnanthes conspicua* Mayer |
| *Achnanthes helvetica* (Hustedt) Lange-Beralot |
| *Achnanthes oblongella* Østrup |
| *Achnanthes ploenensis* Hustedt |
| *Achnanthidium atomus*(Hustedt) Monnier |
| *Achnanthidium catenatum* (Bily & Marvan) Lange-Bertalot |
| *Achnanthidium exiguu* (Grunow) Czarnecki |
| *Achnanthidium latecephalum* Kobayasi |
| *Achnanthidium minutissimum* (Kützing) Czarnecki |
| *Achnanthidium minutissimum* var. *gracillima* (Meister) Bukhtiyarova |
| *Achnanthidium minutissimum* var. *scoticum* (Carter) Cremer |
| *Achnanthidium pyrenaicum* (Hustedt) Kobayasi |
| *Achnanthidium subhudsonis* (Hustdt) Kobayasi |
| *Amphora inariensis* Krammer |
| *Amphora libyca* Ehrenberg |
| *Amphora montana* Krasske |
| *Amphora oligotraphenta* Lange-Bertalot |
| *Amphora pediculus* (Kützing) Grunow |
| *Amphora subturgida* Hustedt |
| *Amphora veneta* Kützing |
| *Anomoeneis sphaerophora* Pfitzer |
| *Aulacoseira ambigua* (Grunow) Simonsen |
| *Bacillaria paradoxa* Gmelin |
| *Cocconeis placentula* Ehrenberg |
| *Cocconeis placentula* var. *euglypta* (Ehrenberg) Grunow |
| *Cocconeis placentula* var. *pseudolineata* (Ehrenberg) Grunow |
| *Craticula cuspidate* (Kützing) Mann |
| *Cyclostephanos invisitatus* (Hohn &Hellerman) Theriot, Stoermer & Hakansson |
| *Cyclotella meneghiniana* Kützing |
| *Cyclotella pseudostelligera* Hustedt |
| *Cyclotella stelligera* Cleve & Grunow |
| *Cymatopleura solea* var*. apiculate* (Smith) Ralts |
| *Cymbella affinis* Kützing |
| *Cymbella aspera* (Ehrenberg) Cleve |
| *Cymbella lanceolata* (Ehrenberg) Van Heurck |
| *Cymbella neoleptoceros* Krammer |
| *Cymbella pusilla* Grunow |
| *Cymbella reichardtii* Krammer |
| *Cymbella silesiaca* Bleisch in Rabenhorst (Encyonema) |
| *Cymbella tumida* (Brebisson) van Heurck |
| *Diadesmis confervacea* Kützing |
| *Diadesmis contenta* (Grunow & van Heurck) Mann |
| *Diatoma vulgaris* Bory |
| *Diploneis elliptica* (Kützing) Cleve |
| *Encyonema minutum* (Hilse in Rabh.) Mann |
| *Encyonema silesiacum* (Bleisch in Rabh.) Mann |
| *Encyonopsis leei* Krammer |
| *Entomoneis alata* |
| *Eolimna subminuscula* |
| *Epithemia adnata* (Kützing) Brebisson |
| *Epithemia reicheltii* Fricke |
| *Epithemia sorex* Kützing |
| *Eunotia exigua* (Brebenhorst) Rabenhorst |
| *Eunotia minor* (Kützing) Grunow in Van Heurck |
| *Eunotia naegelii* |
| *Eunotia pectinalis* var. *recta* Mayer & Patrick |
| *Eunotia polydentula G*runow |
| *Fallacia tenera* (Hustedt) Mann |
| *Fragilaria capucina* var*. vaucheriae* (Kützing) Lange-Bertalot |
| *Fragilaria capucina* Desmazieres |
| *Fragilaria leptostauron* (Ehrenberg) Hustedt |
| *Fragilaria parasitica* (Smith) Grunow |
| *Fragilaria ulna S*ippen angustissima (Grunow) Lange-Bertalot |
| *Fragilaria ulna* var*. acus* (Kützing) Lange-Bertalot |
| *Frustulia rhomboids* (Ehrenberg) Pfitzer |
| *Frustulia rhomboids* var. *viridula* (Brebisson) Cleve |
| *Frustulia sp1* |
| *Frustulia specula* Amosse |
| *Frustulia vulgaris* (Thwaites) De Toni |
| *Gomphonema acuminatum* Ehrenberg |
| *Gomphonema acuminatum* var*. coronata* (Ehrenberg) Smith |
| *Gomphonema affine* Kützing |
| *Gomphonema angustum* (Kützing) Rabenhorst |
| *Gomphonema clavatum* Ehrenberg |
| *Gomphonema clevei* Fricke |
| *Gomphonema gracile* Ehrenberg |
| *Gomphonema lagenula* Maillard |
| *Gomphonema lanceolatum* Agardh |
| *Gomphonema lanceolatum* var. *insignis* |
| *Gomphonema longiceps* Ehrenberg |
| *Gomphonema minutum* (Agardh) Agardh |
| *Gomphonema olivaceum* (Hornemann) Brebisson |
| *Gomphonema parvulum* var. exilissimum Grunow |
| *Gomphonema truncatum* Ehrenberg |
| *Gyrosigma acuminatum* (Kützing) Rabenhorst |
| *Gyrosigma attenuatum* (Kȕtzing) Cleve |
| *Gyrosigma nodiferum* (Grunow) Reimer |
| *Hantzschia amphioxys* (Ehrenberg) Grunow |
| *Luticola goeppertiana* (Bleisch in Rabenhorst) Mann |
| *Luticola mutica* (Kützing) Mann |
| *Luticola ventricosa* (Kützing) Mann |
| *Mayamaea atomus* (Kützing) Lange-Bertalot |
| *Melosira distans* (Ehrenberg) Kützing |
| *Melosira varians* Agardh |
| *Navicula arvensis* Hustedt |
| *Navicula capitata* Ehrenberg |
| *Navicula capitatoradiata* Germain |
| *Navicula cryptocephala* Kützing |
| *Navicula cryptotenella* Lange-Bertalot |
| *Navicula cryptotenelloides* Lange-Bertalot |
| *Navicula dicephala* Ehrenberg |
| *Navicula dicephala* var. *undulata* |
| *Navicula erifuga* Lange-Bertalot |
| *Navicula gregaria* Donkin |
| *Navicula halophila* (Grunow) Cleve |
| *Navicula lanceolata* (Agardh) Ehrenberg |
| *Navicula leptostriata* Jorgensen |
| *Navicula menisculus* Schumann |
| *Navicula minima* Grunow |
| *Navicula molestiformis* Hustedt |
| *Navicula oligotraphenta* Lange-Bertalot & Hofmann |
| *Navicula pelliculosa* Hilsa |
| *Navicula pupula* Kützing |
| *Navicula radiosa* Kützing |
| *Navicula radiosafallax* Lange-Bertalot |
| *Navicula schroeterii* Meister |
| *Navicula schroeterii* var. *symmetrica* (Patrick) Lange-Bertalot |
| *Navicula tenelloides* Hustedt |
| *Navicula trivialis* Lange-Bertalot |
| *Navicula vandamii* Schoeman & Archibald |
| *Navicula veneta* Kützing |
| *Navicula viridula* (Kützing) Ehrenberg |
| *Navicula viridula* var*. rostellata* (Kützing) Cleve |
| *Navicula viridula* var. *germainii* (Wallace) Lange-Bertalot |
| *Nitzschia acicularis* (Kützing) Smith |
| *Nitzschia acuta* Hantzsch |
| *Nitzschia agnita* Hustedt |
| *Nitzschia amphibian* Grunow |
| *Nitzschia amphibioides* Hustedt |
| *Nitzschia angustata* Grunow |
| *Nitzschia aurariae* Cholnoky |
| *Nitzschia capitellata* Hustedt |
| *Nitzschia clausii* Hantzsch |
|  |
| *Nitzschia communis* Rabenhorst |
| *Nitzschia debilis* (Ammott) Grunow |
| *Nitzschia dissipata* (Kützing) Grunow |
| *Nitzschia filiformis* (Smith) van Heurck |
| *Nitzschia flexa* Schumann |
| *Nitzschia frustulum* var. *bulnheimiana* (Rabenhorst) Grunow |
| *Nitzschia graciliformis* Lange-Bertalot & Simonsen |
| *Nitzschia gracilis* Hantzsch |
| *Nitzschia inconspicua* Grunow |
| *Nitzschia intermedia* Hantzsch & Grunow |
| *Nitzschia lacuum* Lange-Bertalot |
| *Nitzschia liebetruthii* Rabenhorst |
| *Nitzschia linearis* (Agardh) Smith |
| *Nitzschia linearis* var. *subtilis* (Grunow) Hustedt |
| *Nitzschia linearis* var. *tenuis* (Smith) Grunow |
| *Nitzschia microcephala* Grunow in Cleve & Moller |
| *Nitzschia nana* Grunow in Van Heurck |
| *Nitzschia palea* (Kützing) Smith |
| *Nitzschia paleacea* Grunow in van Heurck |
| *Nitzschia paleaeformis* Hustedt*.* |
| *Nitzschia perminuta* (Grunow) Peragallo |
| *Nitzschia recta* Hantzsch &Rabenhorst |
| *Nitzschia reversa* Smith |
| *Nitzschia sigma* (Kützing)Smith |
| *Nitzschia sigmoidea* (Nitzsch) Smith |
| *Nitzschia sociabilis* Hustedt |
| *Nitzschia subacicularis* Hustedt |
| *Nitzschia tropica* Hustedt |
| *Nitzschia valdestriata* Aleen & Hustedt |
| *Pinnularia borealis* Ehrenberg |
| *Pinnularia microstauron* var. *brebissonii* (Kützing) Mayer |
| *Pinnularia subcapitata* Gregory |
| *Pinnularia viridiformis* Krammer |
| *Placoneis clementis* (Grunow) Cox |
| *Placoneis elginensis* (Gregory) Cox |
| *Planothidium daui* |
| *Planothidium delicatulum* (Kützing) Round & Bukhtiyarva |
| *Planothidium ellipticum* (Cleve) Round & Bukhtiyarova |
| *Planothidium frequentissimum* (Lange-Bertalot in Krammer & Lange-Bertalot) H. Lange-Bertalot |
| *Planothidium lanceolatum* (Brébisson & Kützing) Lange-Bertalot |
| *Pleurosigma elongatum* Smith |
| *Pseudostaurosira brevistriata* (Grunow. in van Heurck) Williams & Round |
| *Rhoicosphenia abbreviata* (Agardh) Lange-Bertalot |
| *Rhopalodia brebissonii* |
| *Rhopalodia gibberula* (Ehrenberg) Mann |
| *Rhopalodia musculus* (Kützing) Muller |
| *Rhopalodia novae-zealandiae* |
| *Rhopalodia operculata* var*. produdcta* Grunow |
| *Sellaphora seminulum* (Grunow) Mann |
| *Stauroneis anceps* Ehrenberg |
| *Staurophora salina* (Smith) Mereschkowsky |
| *Staurosirella pinnata* (Ehrenberg) Williams & Round |
| *Stenopterobia sp.* |
| *Stephanodiscus neoastraea* Hakansson &Hickel |
| *Surirella angusta* Kützing |
| *Surirella brightwellii* Smith |
| *Surirella ovalis* Brébisson |
| *Tabellaria flocculosa* (Roth) Kützing |
| *Tabularia fasciculate* (Agardh) Williams & Round |
| *Tryblionella apiculata* Gregory |
| *Tryblionella calida* (Grunow in Cleve & Grunow) Mann |
| *Tryblionella debilis* Arnott ex O'Meara |
| *Tryblionella levidensis* Smith |

Appendix 2 List of diatom species in the upper Han River (uHR), China in April 2010.

| *Achnanthes clevei* Grunow |
| --- |
| *Achnanthes helvetica* (Hustedt) Lange-Beralot |
| *Achnanthes oblongella* Østrup |
| *Achnanthidium atomus* (Hust) Monnier |
| *Achnanthidium catenata* (Bily & Marvan) Lange-Bertalot |
| *Achnanthidium minutissimum* (Kützing) Czarnecki |
| *Achnanthidium minutissimum* var. *gracillima* (Meister) Bukhtiyarova |
| *Achnanthidium. minutissimum* var. *scoticum* (Carter) Cremer |
| *Achnanthidium pyrenaicum* (Hustedt) Kobayasi |
| *Achnanthidium subhudsonis* (Hustdt) Kobayasi |
| *Achnanthidium subatomus* (Hustedt) Lange-Bertalot |
| *Amphora inariensis* Krammer |
| *Amphora montana* Krasske |
| *Amphora oligotraphenta* Lange-bertalot |
| *Amphora pediculus* (Kützing) Grunow |
| *Aulacoseira ambigua* (Grunow) Simonsen |
| *Aulacoseira granulate* (Ehrenberg) Simonsen |
| *Caloneis bacillum* (Grunow) Cleve |
| *Cocconeis placentula* var*. euglypta* (Ehrenberg) Grunow |
| *Cocconeis pediculus* Ehrenberg |
| *Craticula accomoda* (Hustedt) Mann |
| *Cyclostephanos dubis* (Fricke) Round |
| *Cyclostephanos invisitatus* (Hohn & Hellerman) Theriot Stoermer & Hakansson |
| *Cyclotella kuetzingiana* |
| *Cyclotella meneghiniana* Kützing |
| *Cyclotella ocellata* Pantocsek |
| *Cyclotella pseudostelligera* Hustedt |
| *Cymatopleura elliptica* (Brebisson) Smith |
| *Cymatopleura solea* (Brebisson) Smith |
| *Cymbella affinis* Kützing |
| *Cymbella cymbiformis* Agardh var. *nonpunctata* Fontell |
| *Cymbella excisa* Kützing |
| *Cymbella microcephala* Grunow |
| *Cymbella naviculiformis* Auerswald & Heib. |
| *Cymbella neoleptoceros* Krammer |
| *Cymbella prostrata* (Berkeley) Grunow (Encyonema) |
| *Cymbella pusilla* Grunow |
| *Cymbella reichardtii* Krammer |
| *Cymbella silesiaca* Bleisch in Rabenhorst (Encyonema) |
| *Cymbella tumida* (Brebisson) van Heurck |
| *Delicata delicatula* |
| *Delicata sinensis* |
| *Denticula tenuis* Kützing |
| *Diatoma vulgaris* Bory |
| *Diploneis elliptica* (Kützing) Cleve |
| *Encyonema minutum* (Hilse in Rabh.) Mann |
| *Encyonopsis leei* Krammer |
| *Encyonopsis leei* Krammervar *sinensis* |
| *Eolimna subminuscula* |
| *Fragilaria capucina Desmazieres* |
| *Fragilaria capucina* var. *vaucheriae* (Kützing) Lange-Bertalot |
| *Fragilaria parasitica* (Smith) Grunow |
| *Fragilaria pinnata* Ehrenbergvar |
| *Fragilaria ulna* Pippen angustissima (Grunow) Lange-Bertalot |
| *Fragilaria ulna* var*. acus* (Kützing) Lange-Bertalot |
| *Frustulia spicula* (Agardh) Cleve |
| *Gomphenema acuminatum* Ehrenberg |
| *Gomphonema angustum* (Kützing) Rabenhorst |
| *Gomphonema clevei* Fricke |
| *Gomphonema minutum* (Agardh) Agardh |
| *Gomphonema olivaceum* (Hornemann) Brebisson |
| *Gomphonema parvulum* Kützing |
| *Gomphonema tergestinum* Fricke |
| *Gyrosigma acuminatum* (Kützing) Rabenhorst |
| *Gyrosigma eximinum* (Thwaites) Boyer |
| *Gyrosigma scalproides* (Rabenhorst) Cleve |
| *Luticola mutica* (Kützing) Mann |
| *Luticola ventricosa* (Kützing) Lange-Bertalot |
| *Mayamaea atomus* (Kützing) Lange-Bertalot |
| *Melosira varians* Agardh |
| *Navicula capitata* Ehrenberg |
| *Navicula capitatoradiata* Germain |
| *Navicula cincta* (Ehrenberg) Ralfs in Pritchard |
| *Navicula cryptocephala* Kützing |
| *Navicula cryptotenella* Lange-Bertalot |
| *Navicula decussis* Oestrup |
| *Navicula dicephala* Ehrenberg |
| *Navicula erifuga* Lange-Bertalot |
| *Navicula gregaria* Donkin |
| *Navicula halophila* (Grunow) Cleve |
| *Navicula menisculus* Schumann |
| *Navicula minima* Grunow |
| *Navicula molestiformis* Hustedt |
| *Navicula oligotraphenta* Lange-Bertalot & Hofmann |
| *Navicula pelliculosa* (Brebisson & Kützing) Hilse |
| *Navicula pupula* Kützing |
| *Navicula radiosa* Kützing |
| *Navicula radiosafallax* Lange-Bertalot |
| *Navicula schroeteri Meister* |
| *Navicula schroeterii*var.*symmetrica* (Patrick) Lange-Bertalot |
| *Navicula trivialis* Lange-Bertalot |
| *Navicula veneta* Kützing |
| *Navicula viridula* (Kützing) Ehrenberg |
| *Navicula viridula* var. *germainii* (Wallace) Lange-Bertalot |
| *Nitzschia acicularis* (Kützing) Smith |
| *Nitzschia agnita* Hustedt |
| *Nitzschia amphibia* Grunow |
| *Nitzschia angustatula* Lange-Bertalot |
| *Nitzschia capitellata* Hustedt |
| *Nitzschia communis* Rabenhorst |
| *Nitzschia dissipata* (Kützing) Grunow |
| *Nitzschia fonticola* Grunow in Cleve & Moller |
| *Nitzschia frustulum* (Kützing) Grunow |
| *Nitzschia graciliformis* Lange-Bertalot & Simonsen |
| *Nitzschia heufleriana* Grunow |
| *Nitzschia incospicua* Grunow |
| *Nitzschia laccum* Lange-Bertalot |
| *Nitzschia liebetruthii* Rabenhorst |
| *Nitzschia linearis* (Agardh) Smith |
| *Nitzschia linearis* (Agardh) Smithvar *subtilis* (Grunow) Hustedt |
| *Nitzschia microcephala* Grunow in Cleve & Moller |
| *Nitzschia nana* Grunow in Van Heurck |
| *Nitzschia palea* (Kützing)Smith |
| *Nitzschia paleaeformis* Hustedt |
| *Nitzschia pussila* (Kützing) Grunow |
| *Nitzschia recta* Hantzsch & Rabenhorst |
| *Nitzschia sinuata* (Thwaites) Grunow var. *delognei* (Grunow) Lange-Bertalot |
| *Nitzschia sinuata* (Thwaites) Grunowvar *tabellaria* Grunow |
| *Nitzschia sociabilis* Hustedt |
| *Planothidium ellipticum* (Cleve) Round & Bukhtiyarova |
| *Planothidium frequentissimum* (Lange-Bertalot in Krammer & Lange-Bertalot) H. Lange-Bertalot |
| *Reimeria uniseriata* Sala Guerrero & Ferrano |
| *Sellaphora bacillum* (Ehrenberg) Mann |
| *Sellaphora seminulum* (Grunow) Mann |
| *stephanodiscus neoastraea* Hakansson & Hickel |
| *Surirella angusta* Kützing |
| *Surirella brebissonii* Krammer & Lange-Bertalot |
| *Surirella linearis* Smith var. *helvetica* (Brunow) Meister |
| *Surirella minuta* Brebisson |
| *Surirella suecica* Grunow |
| *Surirella terricola* Lange-Bertalot & Alles |
| *Tryblionella apiculata* Gregory |
